# Lenticulostriate Arteries and Basal Ganglia Changes in Cerebral Autosomal Dominant Arteriopathy With Subcortical Infarcts and Leukoencephalopathy, a High-Field MRI Study

**DOI:** 10.3389/fneur.2019.00870

**Published:** 2019-08-09

**Authors:** Chen Ling, Xiaojing Fang, Qingle Kong, Yunchuang Sun, Bo Wang, Yan Zhuo, Jing An, Wei Zhang, Zhaoxia Wang, Zihao Zhang, Yun Yuan

**Affiliations:** ^1^Department of Neurology, Peking University First Hospital, Beijing, China; ^2^Department of Neurology, Peking University International Hospital, Beijing, China; ^3^State Key Laboratory of Brain and Cognitive Science, Beijing MR Center for Brain Research, Institute of Biophysics, Chinese Academy of Sciences, Beijing, China; ^4^CAS Center for Excellence in Brain Science and Intelligence Technology, Beijing, China; ^5^University of Chinese Academy of Sciences, Beijing, China; ^6^Siemens Shenzhen Magnetic Resonance Ltd., Shenzhen, China

**Keywords:** cerebral autosomal dominant arteriopathy with subcortical infarcts and leukoencephalopathy (CADASIL), 7.0-Tesla magnetic resonance imaging (7.0-T MRI), time-of-flight-magnetic resonance angiography (TOF-MRA), lenticulostriate arteries, basal ganglia

## Abstract

**Background and Purpose:** Cerebral autosomal dominant arteriopathy with subcortical infarcts and leukoencephalopathy (CADASIL) mainly affects the cerebral small arteries. We aimed to analyze changes in the lenticulostriate arteries (LSAs) and the basal ganglia in patients with CADASIL using high-field magnetic resonance imaging (7.0-T MRI).

**Methods:** We examined 46 patients with CADASIL and 46 sex- and age-matched healthy individuals using 7.0-T MRI. The number and length of the LSAs, and the proportion of discontinuous LSAs were compared between the two groups. The Mini-Mental State Examination score, the modified Rankin Scale, the Barthel Index, and the MRI lesion load of the basal ganglia were also examined in patients with CADASIL. We analyzed the association between LSA measurements and the basal ganglia lesion load, as well as the association between LSA measurements and clinical phenotypes in this patient group.

**Results:** We observed a decrease in the number of LSA branches (*t* = −2.591, *P* = 0.011), and an increase in the proportion of discontinuous LSAs (*z* = −1.991, *P* = 0.047) in patients with CADASIL when compared with healthy controls. However, there was no significant difference in the total length of LSAs between CADASIL patients and healthy individuals (*t* = −0.412, *P* = 0.682). There was a positive association between the number of LSA branches and the Mini-Mental State Examination scores of CADASIL patients after adjusting for age and educational level (β = 0.438; 95% CI: 0.093, 0.782; *P* = 0.014). However, there was no association between LSA measurements and the basal ganglia lesion load among CADASIL patients.

**Conclusions:** 7.0-T MRI provides a promising and non-invasive method for the study of small artery damage in CADASIL. The abnormalities of small arteries may be related to some clinical symptoms of CADASIL patients such as cognitive impairment. The lack of association between LSA measurements and the basal ganglia lesion load among the patients suggests that changes in the basal ganglia due to CADASIL are caused by mechanisms other than anatomic narrowing of the vessel lumen.

## Introduction

Cerebral autosomal dominant arteriopathy with subcortical infarcts and leukoencephalopathy (CADASIL) is an inherited small vessel disease caused by mutations in the *NOTCH3* gene (1, 2). The main clinical features of CADASIL include recurrent transient ischemic attack (TIA) and ischemic stroke, migraine with or without aura, progressive cognitive decline, and mood disturbances (3–5). Granular osmiophilic material (GOM) deposits in the basement membrane of vascular smooth muscle cells (VSMCs) represent the pathological hallmark of CADASIL (1, 6). Magnetic resonance imaging (MRI) also plays a crucial role in the diagnosis and clinical evaluation of CADASIL. Diffuse white matter hyperintensities (WMHs), multiple lacunar infarctions (LIs), and cerebral microbleeds (CMBs) are the typical MRI abnormalities in patients with CADASIL (7, 8).

Small arteries, especially the cerebral small arteries are mainly affected in CADASIL. The lenticulostriate arteries (LSAs) are the major cerebral small arteries supplying blood to the basal ganglia, a region of the brain that is particularly susceptible in CADASIL (9, 10). Ultrastructural analysis is the commonly used method for studying changes of small cerebral arteries in CADASIL. Investigations using such methods have revealed that small cerebral arteries in patients with CADASIL exhibited significantly thickened vessel walls, which contain deposits of various collagen and extracellular matrix proteins (11–13). However, it is difficult to conduct large-scale histopathological investigations to more fully elucidate the changes of small cerebral arteries, such as the LSAs in CADASIL, because of the limitations in obtaining post-mortem brain samples. Magnetic resonance angiography (MRA) provides an effective, non-invasive method for observing cerebral blood vessels *in vivo*. However, because of the limitations in signal-to-noise ratio, traditional 3.0-T MRA is incapable of visualizing the intracranial small arteries. Recently, several studies have confirmed the superiority of 7.0-T time-of-flight MRA (TOF-MRA) for examining the intracranial small arteries, especially the LSAs (14–16), providing a powerful tool for the study of CADASIL arteriopathy.

In the present study, we aimed to examine changes of the LSAs and the basal ganglia in patients with CADASIL using 7.0-T MRI, and to analyze the association between LSA measurements and the basal ganglia lesion load, as well as the association between LSA measurements and clinical phenotypes in this patient population.

## Materials and Methods

### Patients

The present study was approved by the institutional review board and ethics committee at the Peking University First Hospital, and the study was conducted in accordance with the ethical standards laid down in the 1964 Declaration of Helsinki and its later amendments. Fifty patients with CADASIL and 53 sex- and age-matched healthy controls were recruited and examined after obtaining written informed consent. The diagnosis of CADASIL was based on the gene sequencing results. The positive gene result was defined as the presence of a heterozygous missense mutation, which is pathogenic according to previous studies, in the *NOTCH3* gene. If GOM deposits on the basement membrane of VSMCs were found in skin biopsy of the patient, the gene results were considered positive, even though the mutation was not previously reported (5). Healthy controls had no known cerebrovascular disease or related risk factors (e.g., TIA, stroke, diabetes, hypertension, dyslipidemia, cardiac disease, psychiatric illness, major head trauma, or Alzheimer's disease), as confirmed via clinical interviews and examinations. Eleven of the controls admitted to being current or former smokers and 13 controls admitted to alcohol consumption.

The following clinical and demographic data were collected for each patient at the time of inclusion: age, sex, disease duration (determined based on the first occurrence of neurological symptoms), history of hypertension (defined as blood pressure at the time of presentation (≥140/90 mmHg) or previous diagnosis of hypertension), history of diabetes (defined by previous diagnosis), history of hyperlipidemia (defined by previous diagnosis), and history of smoking/alcohol consumption (defined as those who are currently consuming alcohol/smoking tobacco at least once a week, or those who have quit smoking or drinking less than a year ago). We also recorded the clinical symptoms of the patients, such as TIA/stroke, cognitive impairment, etc. Patients with cognitive impairment were defined as those whose Mini-Mental State Examination (MMSE) scores were lower than the lower quartile of the age- and educational level-matched healthy controls (17). The degrees of dependence of all patients were determined using the modified Rankin Scale (mRS) and the Barthel Index (BI) (18).

### Brain MRI Analysis

All patients underwent MRI examination using a 7.0-T whole-body research MR system (Siemens Healthineers, Erlangen, Germany). The following imaging sequences were included in the scanning: T1-weighted (T1w) magnetization-prepared rapid gradient-echo for the localization and the identification of LIs, 3-dimensional (3D) high-resolution TOF-MRA for displaying the LSAs, T2-weighted (T2w) fluid-attenuated inversion recovery (FLAIR) for identifying the WMHs and LIs, and susceptibility weighted imaging (SWI) for detecting CMBs. The imaging parameters of the sequences are summarized in Supplementary Table 1.

3D reconstruction and analysis of MRA images were performed using a non-commercial software (Horos^®^ *https://horosproject.org*) (19, 20). Firstly, we examined the whole circle of Willis to exclude the presence of structural abnormalities in large vessels. We excluded the imaging data from three patients and seven controls because of poor image quality caused by head motion, and moreover, excluded the data from one patient because of a history of head trauma. Finally, 46 patients and 46 sex- and age-matched healthy controls were included.

We then counted the number of stems and branches of the LSAs derived from the first segment of the bilateral anterior cerebral arteries (ACAs) and from the first segment of the bilateral middle cerebral arteries (MCAs). Only the blood vessels pointing toward the anterior perforated substances were counted. Stems were defined as LSAs that originated directly from the ACAs or MCAs. The branches were defined as daughter vessels originating from the parent LSA stems, without any subordinate branches (single vessels) (21, 22). If the trunk had no branches, it was recorded as both stem and branch (Supplementary Figure 1). Secondary outcome measures included the maximum length of the LSAs and the proportion of discontinuous LSAs in each participant. To measure the maximum length of the LSAs, maximum intensity projections (MIPs) were reconstructed for coronal slabs (thickness: 28 mm) using Horos, and the lengths of the LSAs were determined as the straight axial distance from the highest point of the middle cerebral artery to the end of the longest perforating artery (23, 24). We used the total length of the left and right LSAs in the final data analysis. To evaluate discontinuous LSAs, we first identified arteries with signal interruption on coronal MIP images. We then returned to the axial image to identify and measure the contrast-to-noise ratio (CNR) of the vessel lumen. The CNR was calculated by $\frac{\text{mean }\left(\text{signa}{\text{l}}_{\text{lumen}}\right)}{\text{mean }\left(\text{signa}{\text{l}}_{\text{tissue}}\right)}$ (25). The same region-of-interest was selected to obtain the CNR of 20 arteries. CNR in the normal signal region of arteries was 2.38 ± 0.58, while that in areas with interrupted signal was 1.37 ± 0.31. Discontinuous LSAs were defined as arteries with more than one region with CNR <1.7, and the proportion of discontinuous LSAs was calculated by $\frac{\text{number of discontinuous LSAs}}{\text{total number of LSAs}}$.

**Figure 1:**
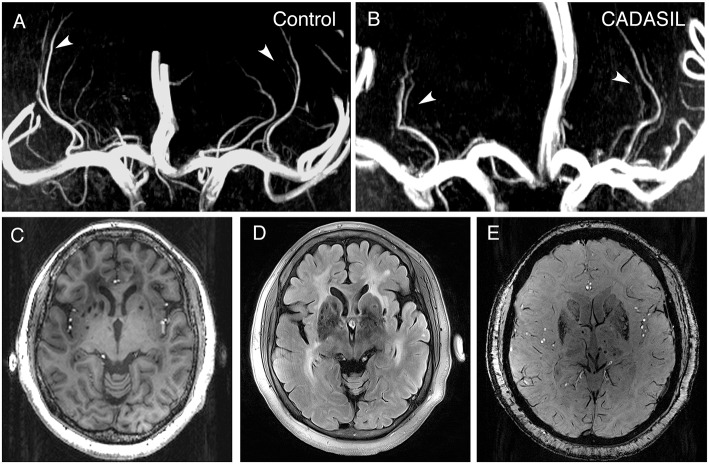
7.0-T MRI images of patients with CADASIL. **(A)** Representative LSA image of a 37-year-old healthy woman obtained using 7.0-T TOF-MRA. Arrows indicate bilateral arteries. **(B)** Representative LSA image of a 39-year-old woman with CADASIL obtained using 7.0-T TOF-MRA. Arrows indicate bilateral arteries. Compared to healthy control, decreased number of LSAs and increased proportion of discontinuous LSAs can be observed. **(C)** T1-weighted sequence of a 51-year-old woman with CADASIL demonstrating LIs in the basal ganglia. **(D)** FLAIR sequence of the same patient displaying WMHs and LIs in the basal ganglia. **(E)** SWI sequence of the same patient exhibiting CMBs. LSA, lenticulostriate artery; LIs, lacunar infarctions; WMHs, white matter hyperintensities; CMBs, cerebral microbleeds.

Circular or elliptical lesions with a diameter of 3–15 mm, with a surrounding rim of high signal intensity on the FLAIR sequence, and with the same signal as the cerebrospinal fluid on both T1 and FLAIR sequence were defined as LIs; WMH was defined as a high signal intensity region with a diameter ≥5 mm on FLAIR sequence; Circular lesions with a diameter of 2–10 mm on SWI sequence were defined as CMBs (26). The number of LIs and CMBs on the right and left sides of the basal ganglia region (including the basal ganglia, and the internal and external capsule) were counted manually. The WMH load for the right and left sides of the basal ganglia region was measured using the basal ganglia subscale of the age-related white matter change (ARWMC) scores [Supplemental Table 2; (27)]. The basal ganglia lesions of a 51-year-old patient, as well as the LSAs of a 39-year-old patient and an age- and sex-matched control are shown in Figure 1.

The number and length of LSAs, as well as the proportion of discontinuous LSAs were examined by a clinician and a radiologist (LC and KQL), and the MRI lesions were assessed by two clinicians (LC and FXJ). The mean of the measurements was used for final statistical analyses. The intraclass correlation coefficient (ICC) for the number of LSA stems of healthy controls was 0.769 [95% confidence interval (CI): 0.618–0.865], that for the number of LSA branches of healthy controls was 0.873 (95% CI: 0.782–0.928), that for the number of LSA stems of CADASIL patients was 0.854 (95% CI: 0.750–0.916), and that for the number of LSA branches of CADASIL patients was 0.867 (95% CI: 0.773–0.924). The ICC for the length of LSAs of healthy controls was 0.992 (95% CI: 0.985–0.995), that for the length of LSAs of CADASIL patients was 0.990 (95% CI: 0.981–0.994), that for the proportion of discontinuous LSAs of healthy controls was 0.873 (95% CI: 0.782–0.928), and that for the proportion of discontinuous LSAs of CADASIL patients was 0.840 (95% CI: 0.729–0.908). Among patients with CADASIL, the ICC for ARWMC scores of the basal ganglia was 0.955 (95% CI: 0.920–0.975), that for number of LIs in the basal ganglia was 0.869 (95% CI: 0.775–0.925), and that for number of CMBs in the basal ganglia was 0.990 (95% CI: 0.982–0.994).

### Statistical Analysis

Statistical analyses were performed using SPSS version 20.0 (SPSS Inc., Chicago, IL, USA). The normality of the data was analyzed using the Kolmogorov-Smirnov test. Normally distributed data were compared using independent two samples *t*-tests (*t*), whereas non-normally distributed data were compared using Mann–Whitney *U*-tests (*z*). When grouping by history of smoking/alcohol consumption, all data were analyzed using non-parametric tests due to the small sample size, and the *P*-value was Bonferroni corrected (Bonferroni corrected *P* = P^*^2). *Chi*-square tests were used to compare the ratios.

We performed univariate analyses using Spearman rank correlation and Mann–Whitney *U*-tests. Adjustments were achieved using multiple linear regression (for ARWMC scores and MMSE scores), or logistic regression (for the presence of LIs and the presence of CMBs). ARWMC scores for the basal ganglia, the presence of LIs in the basal ganglia, the presence of CMBs in the basal ganglia, and MMSE scores were used as dependent variables. Age and LSA measurements, or age and educational level were included as independent variables in the final regression model. Because of the small number of patients with hypertension, diabetes, or hyperlipidemia, we did not include these variables in the final analyses. The ICC was analyzed using a two-way random effects model. Statistical significance was defined as *P* < 0.05.

## Results

### Clinical Manifestations of Patients With CADASIL

As shown in Table 1, among the 46 patients with CADASIL, 38 were symptomatic (45.16 ± 8.90 years; range, 28–63 years), while eight were asymptomatic (34.25 ± 6.52 years; range, 23–43 years). Hypertension, diabetes, and hyperlipidemia were noted in six, two, and six symptomatic patients, respectively. Thirteen symptomatic patients reported a history of smoking, while 17 reported a history of alcohol consumption. Among the asymptomatic patients, with the exception of two patients with a history of smoking and one patient with a history of alcohol consumption, there were no other risk factors for cerebrovascular disease. The mean age at the onset of CADASIL, among symptomatic patients, was 39.53 ± 7.95 years (range, 24–56 years), and the median duration of disease was 5.5 years (range, 0–17 years). There were 35 patients had a history of TIA/stroke and nine patients suffered from cognition impairment.

**Table 1:** Demographic and clinical features of patients with CADASIL.

**Category**	**Patients with CADASIL**		**Healthy controls**
	**Symptomatic (*n* = 38)**	**Asymptomatic (*n* = 8)**	
Age (mean ±*SD*; range)	45.16 ± 8.90 (28–63)	34.25 ± 6.52 (23–43)	41.13 ± 10.26 (25–64)
Age of onset (mean ±*SD*; range)	39.53 ± 7.95 (24–56)	–	–
Disease duration (median; range)	5.5 (0–17)	–	–
Sex (male/female)	21/17	3/5	24/22
History of smoking	13/38	2/8	11/46
History of alcohol consumption	17/38	1/8	13/46
Hypertension	6/38	0/8	0/46
Diabetes mellitus	2/38	0/8	0/46
Hyperlipidemia	6/38	0/8	0/46
Cognitive impairment	9/31	0/4	–
TIA/stroke	35/38	0/8	–

*MRI, magnetic resonance imaging; CADASIL, cerebral autosomal dominant arteriopathy with subcortical infarcts and leukoencephalopathy; TIA, transient ischemia attack*.

### Changes in LSAs in Patients With CADASIL

There was no significant difference in age (*t* = 1.036, *P* = 0.303) between the CADASIL patients and healthy controls. The number of LSA branches was lower in patients than in controls (*t* = −2.591, *P* = 0.011), whereas no significant difference in the number of stems was found between the groups (*z* = −1.617, *P* = 0.106). An increased proportion of discontinuous LSAs was also observed in patients with CADASIL (*z* = −1.991, *P* = 0.047). However, there was no significant difference in the total length of LSAs between the patients and healthy individuals (*t* = −0.412, *P* = 0.682) (Table 2).

**Table 2:** Comparison of the measurements of lenticulostriate arteries between CADASIL patients and healthy controls.

**Category**	**Patients with CADASIL (*n* = 46)**	**Healthy controls (*n* = 46)**	***t (z)***	**95% CI (p25, p75)**	***P***
Age (years)^a^	43.26 ± 9.45	41.13 ± 10.26	1.036	−1.955, 6.215	0.303
Sex (male/female)^c^	24/22	24/22	–	–	1.000
LSA stems (number)^b^	6.00 (6.00, 7.00)	7.00 (6.50, 7.50)	−1.617	5.500, 8.000	0.106
LSA branches (number)^a^	10.72 ± 2.85	12.22 ± 2.70	−2.591	−2.650, −0.350	0.011^*^
Proportion of discontinuous LSAs^b^	7.29 (4.17, 13.27)	0.00 (0.00, 7.84)	−1.991	0.000, 15.846	0.047^*^
Length of LSAs (mm)^a^	54.95 ± 6.85	55.56 ± 7.41	−0.412	−3.568, 2.343	0.682

*LSAs, lenticulostriate arteries; Age, number of LSA branches and length of LSAs are presented as mean ± SD. Number of LSA stems and proportion of discontinuous LSAs are presented as median with 95% CI*.

* *Significant difference*.

a *Independent two samples t-test, and 95% CI of the difference is shown*.

b *Mann-Whitney U-test, and p25–p75 of the transformation rank is shown*.

c *Chi-square test*.

**Figure 2:**
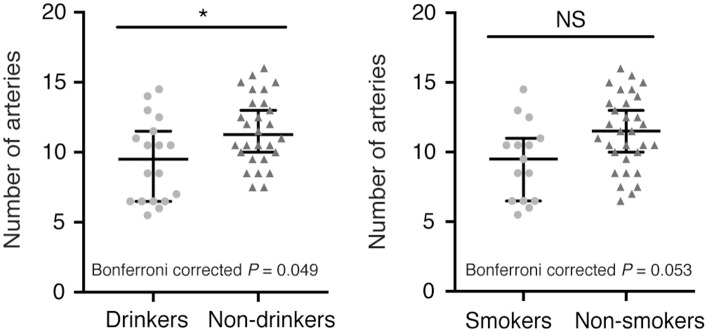
Comparison of the number of LSA branches between patients with or without a history of smoking/alcohol consumption. (Left) Drinkers vs. non-drinkers. (Right) Smokers vs. non-smokers. Data are presented as the median with the 95% CI. NS, not significant; ^*^Bonferroni corrected *P* < 0.05. LSA, lenticulostriate artery.

In addition, we observed fewer LSA branches in patients with a history of alcohol consumption (*z* = −2.247, Bonferroni corrected *P* = 0.049) than in those without such a history, but we did not find a significant change in the number of LSA branches in patients with a history of smoking (*z* = −2.221, Bonferroni corrected *P* = 0.053) (Figure 2). There was no significant difference in the proportion of discontinuous LSAs between smokers/drinkers and non-smokers/non-drinkers (smokers vs. non-smokers: *z* = −1.145, Bonferroni corrected *P* = 0.504; drinkers vs. non-drinkers: *z* = −0.034, Bonferroni corrected *P* = 1.946). Moreover, there was no significant difference in the total length of LSAs between smokers/drinkers and non-smokers/non-drinkers (smokers vs. non-smokers: *z* = −1.019, Bonferroni corrected *P* = 0.616; drinkers vs. non-drinkers: *z* = −1.305, Bonferroni corrected *P* = 0.384).

### Association Between LSA Measurements and Basal Ganglia Lesion Load of CADASIL Patients

In CADASIL patients, we did not find an association between LSAs measurements and the basal ganglia lesion load in univariate analyses. However, we found that patients with a history of alcohol consumption had more CMBs (*z* = −2.026; *P* = 0.043) and more LIs (*z* = −2.424; *P* = 0.015) in the basal ganglia (Supplementary Table 3).

After adjusting for age, there was no significant association between the number of LSAs and ARWMC scores (β = −0.049; 95% CI: −0.219, 0.121; *P* = 0.565) for the basal ganglia, the presence of LIs (*OR* = 0.878; 95% CI: 0.698, 1.103; *P* = 0.264) in the basal ganglia, or the presence of CMBs (*OR* = 1.011; 95% CI: 0.799, 1.279; *P* = 0.927) in the basal ganglia in CADASIL patients. Moreover, we observed no significant association between the proportion of discontinuous LSAs and ARWMC scores (β = 0.002; 95% CI: −0.032, 0.035; *P* = 0.912) for the basal ganglia, the presence of LIs (*OR* = 1.041; 95% CI: 0.988, 1.098; *P* = 0.134) in the basal ganglia, or the presence of CMBs (*OR* = 0.982; 95% CI: 0.932, 1.035; *P* = 0.500) in the basal ganglia in the patient group after adjusting for age. There was also no significant association between the length of LSAs and ARWMC scores (β = −0.030; 95% CI: −0.099, 0.039; *P* = 0.387) for the basal ganglia, the presence of LIs (*OR* = 0.980; 95% CI: 0.895, 1.073; *P* = 0.665) in the basal ganglia, or the presence of CMBs (*OR* = 0.951; 95% CI: 0.860, 1.051; *P* = 0.326) in the basal ganglia in the patient group after adjusting for age (Table 3). However, after adjusting for age and LSAs measurements, alcohol consumption increased the risk of CMBs and LIs in the basal ganglia in the patient group (CMBs: *OR* = 6.000; 95% CI: 1.472, 24.454; *P* = 0.012; Nagelkerke *R*^2^ = 0.199) (LIs: OR = 6.299; 95% CI: 1.394, 28.456; *P* = 0.017; Nagelkerke *R*^2^ = 0.278).

**Table 3:** Age adjusted association between LSA measurements and MRI lesion load of the basal ganglia in CADASIL patients.

**Variables**	**ARWMC scores**^**a**^			**Presence of LIs**^**b**^			**Presence of CMBs**^**b**^		
	**β**	**95% CI**	***P***	**OR**	**95% CI**	***P***	**OR**	**95% CI**	***P***
Number of LSA branches	−0.049	−0.219, 0.121	0.565	0.878	0.698, 1.103	0.264	1.011	0.799, 1.279	0.927
Proportion of discontinuous LSAs	0.002	−0.032, 0.035	0.912	1.041	0.988, 1.098	0.134	0.982	0.932, 1.035	0.500
Length of LSAs	−0.030	−0.099, 0.039	0.387	0.980	0.895, 1.073	0.665	0.951	0.860, 1.051	0.326

*LSAs, lenticulostriate arteries; LIs, lacunar infarctions; ARWMC, age-related white matter change; CMBs, cerebral microbleeds; CI, confidence interval*.

a *Multivariate linear regression analysis (“enter” model)*.

b *Binary logistic regression (“enter” model)*.

### Association Between LSA Measurements and Clinical Phenotypes of CADASIL Patients

Using univariate analysis, we found an association between the number of LSA branches and MMSE scores of CADASIL patients (ρ = 0.413; *P* = 0.014). However, no significant association was found between the number of LSA branches and the mRS/BI scores (mRS scores: ρ = −0.142, *P* = 0.348; BI scores: ρ = 0.039, *P* = 0.799). The proportion of discontinuous LSAs and the length of the LSAs were not significantly associated with all the three clinical scores of the patients (Table 4). After adjusting for age and educational level, there was still a positive association between the number of LSA branches and MMSE scores in CADASIL patients (β = 0.438; 95% CI: 0.093, 0.782; *P* = 0.014). No significant difference in LSA measurements was found between patients with a history of TIA/stroke and patients without a history of TIA/stroke (the number of LSA branches: *z* = −0.478, *P* = 0.633; the length of LSAs: *z* = −0.167, *P* = 0.867; the proportion of discontinuous LSAs: *z* = −1.363, *P* = 0.173).

**Table 4:** Association between LSA measurements and clinical phenotypes of CADASIL patients.

**Variables**	**MMSE scores**		**mRS scores**		**BI scores**	
	**ρ**	***P***	**ρ**	***P***	**ρ**	***P***
Number of LSA branches	0.413	0.014^*^	−0.142	0.348	0.039	0.799
Proportion of discontinuous LSAs	−0.056	0.751	0.277	0.062	−0.076	0.617
Length of LSAs	0.183	0.294	−0.109	0.472	0.056	0.711

*LSAs, lenticulostriate arteries; MMSE, Mini-Mental State Examination; mRS, modified Rankin Scale; BI, Barthel Index*.

* *Significant difference. Data were analyzed using Spearman rank correlation (ρ)*.

## Discussion

In the present study, we aimed to analyze the changes in LSAs among patients with CADASIL using a 7.0-T MRI. Our findings indicate that patients with CADASIL exhibit fewer LSA branches and a higher proportion of discontinuous LSAs than healthy individuals. Although there was no association between the measurements of LSAs and the basal ganglia lesion load in patients with CADASIL, we observed a positive association between the number of LSA branches and MMSE scores in CADASIL patients.

Clinical manifestations and MRI features in our patients were consistent with those previously reported in Chinese CADASIL cohorts (3, 5, 28). The median number and length of LSAs in our cohort of healthy individuals were similar to that observed in previous studies (14, 23, 24), confirming the reliability of our imaging strategies. We observed that alcohol consumption aggravated damage to the LSAs, consistent with the well-known harmful effects of drinking on the cerebrovascular system. Therefore, controlling alcohol intake is especially important for patients with CADASIL. Presently, our results did not show a significant decrease in the number of LSAs in patients with a history of smoking. However, as the Bonferroni corrected *P*-value is close to 0.05 (Bonferroni corrected *P* = 0.053), we presume that further increase in sample size may lead to a significant decrease in the number of LSAs in patients with a history of smoking.

In the present study, patients with CADASIL exhibited a decrease in the number of LSA branches and an increase in the proportion of discontinuous LSAs. Since TOF-MRA is based on the in-flow effect of cerebral blood flow, these results suggested that there was an interruption in blood flow. Some post-mortem studies have indicated that patients with CADASIL exhibit stenosis and occlusion of the cerebral small arteries (29, 30), whereas other studies have suggested abnormalities in hemodynamics and vasomotor activities in both patients with CADASIL and mouse models of CADASIL (31–33). Because of the small luminal diameter of LSA branches and the limited resolution of MRA devices, decreases in blood flow caused by stenosis or hemodynamic changes may go undistinguished and manifest as decreased number or discontinuity of arteries on MRA images. Further studies are required to determine which of these two factors plays a major role. A previous study involving 22 patients with CADASIL and 11 healthy controls reported that there were no changes in the number of LSAs in patients with CADASIL (23), inconsistent with our findings. Because our study included 46 patients as well as 46 age- and sex-matched controls, these discrepancies may be related to the differences in sample size. Thus, 7.0-T MRI is a promising and non-invasive method for the study of small artery damage in CADASIL, which may aid evaluation of the clinical condition of CADASIL patients in the future.

In our study, there was no association between LSA measurements and the basal ganglia lesion load, consistent with the findings of a previous 7.0-T MRI-based study on patients with CADASIL (23). LIs and WMHs are usually thought to be caused by hypoperfusion, which can also be attributed to hemodynamic abnormalities other than arterial stenosis. Additional studies have suggested that WMHs can also be attributed to a dysfunctional blood-brain barrier (34, 35). Indeed, hemodynamic abnormalities and dysfunctional blood-brain barrier have been observed in studies involving both patients with CADASIL and animal models of CADASIL (31, 33, 36, 37). Thus, we speculate that basal ganglia lesions in patients with CADASIL may be caused by hemodynamic abnormalities or a dysfunctional blood-brain barrier. This may explain why we were unable to identify an association between LSA changes and the basal ganglia lesion load. In addition, although the resolution of 7.0-T MRA has improved, it is still impossible to observe vessels with diameter <250 μm by *in vivo* imaging (23). Therefore, the possibility that the stenosis of the lumen of smaller vessels leads to the lesions of the basal ganglia cannot be ruled out. It is also possible that the number of patients in our study was too small to yield a significant association. Further, we observed that alcohol consumption significantly increased the risk of CMBs and LIs in the basal ganglia in the patient group, highlighting the importance of controlling alcohol intake among patients with CADASIL.

In addition, we found a positive association between the number of LSA branches and MMSE scores in CADASIL patients, suggesting that abnormalities of small arteries may be related to some clinical symptoms of CADASIL patients. There may be two explanations for this association. Firstly, in recent years, the importance of the basal ganglia in cognition has been reported by many studies, and it is known to participate in several cognitive pathways such as executive function, procedural memory, and attention (38, 39). Studies of type 1 diabetes have suggested that reduced cerebral blood flow in the bilateral caudate nucleus-thalamus is associated with abnormal executive function (39). Therefore, the impaired blood supply and basal ganglia dysfunction caused by LSA abnormalities in CADASIL patients may directly lead to cognitive impairment. Secondly, the LSAs are a part of the cerebral perforating artery system, and therefore the LSA abnormalities we observed may indirectly reflect changes to the whole cerebral perforating artery system. Abnormal cerebral perfusion and brain tissue damage caused by changes to the cerebral perforating artery system could further lead to cognitive impairment in CADASIL patients. However, the above hypothesis lacks direct evidence and needs further research to confirm its validity.

The present study possesses several limitations of note. Because CADASIL is a rare disease, our analysis is inherently limited by weaknesses in the case-control study design, including imperfect matching, inevitable recall bias, and difficulty in determining causal relationships. In addition, the method for measuring the length of LSAs was simplified because of the lack of a software for tracking and reconstructing LSAs. Although this modified method may reflect the extensive stenosis of LSAs, such measurements are easily affected by the curvature of blood vessels and may not reflect the true length of these vessels.

## Conclusions

We have shown that patients with CADASIL exhibit fewer LSA branches and a higher proportion of discontinuous LSAs than healthy individuals when examined using 7.0-T MRI. This suggests that 7.0-T MRI is a promising and non-invasive method for the study of small artery damage in CADASIL. The abnormalities of small arteries may be related to some of the clinical symptoms of CADASIL patients such as cognitive impairment. However, since we observed no association between the LSA measurements and the basal ganglia lesion load, the changes in the basal ganglia due to CADASIL are most likely caused by mechanisms other than the anatomic narrowing of the vessel lumen, such as hemodynamic abnormalities or a dysfunctional blood-brain barrier.

## Data Availability

The raw data supporting the conclusions of this manuscript will be made available by the authors, without undue reservation, to any qualified researcher.

## Ethics Statement

The present study was approved by the institutional review board and ethics committee at Peking University First Hospital and has been performed in accordance with the ethical standards laid down in the 1964 Declaration of Helsinki and its later amendments. Fifty patients with CADASIL and 53 age-matched healthy controls were recruited and examined after obtaining written informed consent.

## Author Contributions

YY and ZZ contributed conception and design of the study. CL and XF collected and organized the database, performed the MRI analysis and the statistical analysis. CL and QK performed the MRI analysis. CL wrote the first draft of the manuscript. QK, YS, WZ, and ZW wrote sections of the manuscript. BW, YZ, and JA participated in the design and improvement of MRI scan sequences. All authors contributed to manuscript revision, read, and approved the submitted version.

### Conflict of Interest Statement

JA was employed by Siemens Shenzhen company. The remaining authors declare that the research was conducted in the absence of any commercial or financial relationships that could be construed as a potential conflict of interest.

## Abbreviations

ARWMC: age-related white matter change
CADASIL: cerebral autosomal dominant arteriopathy with subcortical infarcts and leukoencephalopathy
CI: confidence interval
CMBs: cerebral microbleeds
CNR: contrast-to-noise ratio
ICC: intraclass correlation coefficient
LIs: lacunar infarctions
LSAs: lenticulostriate arteries
MMSE: Mini-Mental State Examination
MRI: magnetic resonance imaging
TIA: transient ischemic attack
TOF-MRA: time-of-flight magnetic resonance angiography
WMH: white matter hyperintensity.
