# Supplementary material for: Lenticulostriate Arteries and Basal Ganglia Changes in Cerebral Autosomal Dominant Arteriopathy With Subcortical Infarcts and Leukoencephalopathy, a High-Field MRI Study
Source: Front Neurol. 2019 Aug 9;10:870. doi: 10.3389/fneur.2019.00870 (PMC6696621; doi:10.3389/fneur.2019.00870)
Supplement: Supplementary file 1 [file Data_Sheet_1.PDF]

Supplementary Material

Lenticulostriate Arteries and Basal Ganglia Changes in Cerebral Autosomal Dominant Arteriopathy with Subcortical Infarcts and Leukoencephalopathy, A High-Field MRI study

Chen Ling[1#], Xiaojing Fang[1,2#], Qingle Kong[3,4,5], Yunchuang Sun[1], Bo Wang[3,4,5], Yan Zhuo[3,4,5], Jing An[6], Wei Zhang[1], Zhaoxia Wang[1], Zihao Zhang[3,4,5*], Yun Yuan[1*]

[1]Department of Neurology, Peking University First Hospital, Beijing, China

[2]Department of Neurology, Peking University International Hospital, Beijing, China

[3]State Key Laboratory of Brain and Cognitive Science, Beijing MR Center for Brain Research, Institute of Biophysics, Chinese Academy of Sciences, Beijing, China

[4]CAS Center for Excellence in Brain Science and Intelligence Technology, Beijing, China

[5]University of Chinese Academy of Sciences, Beijing, China

[6]Siemens Shenzhen Magnetic Resonance Ltd., Shenzhen, China

[#] These authors contributed equally to this work.

*** Correspondence:**

Yun Yuan, MD, PhD. Department of Neurology, Peking University First Hospital. E-mail: yuanyun2002@126.com.

Zihao Zhang, PhD. State Key Laboratory of Brain and Cognitive Science, Beijing MR Center for Brain Research, Institute of Biophysics, Chinese Academy of Sciences. E-mail: zhzhang@ibp.ac.cn.

Supplementary Methods

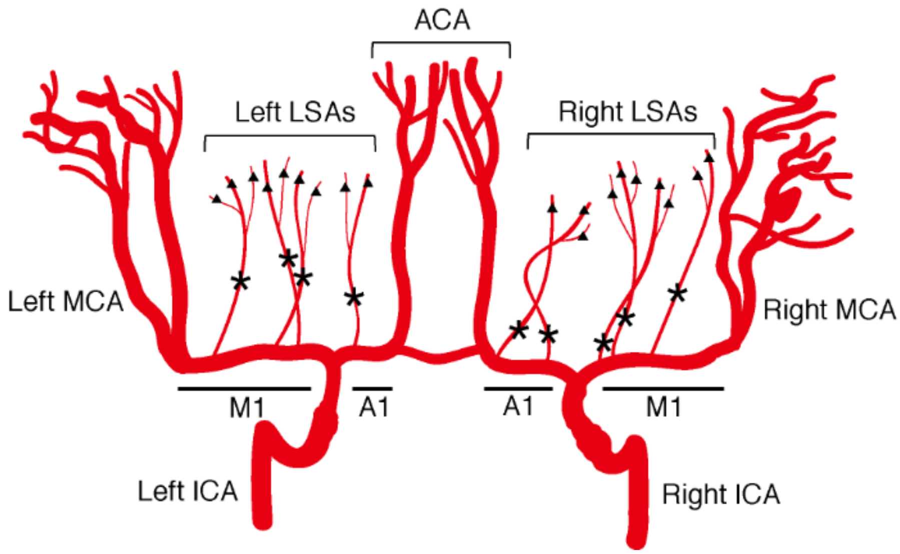

Supplementary Figure 1. A schematic diagram of lenticulostriate arteries. LSAs are arterioles originating from the bilateral MCA and ACA, supplying the basal ganglia. Stems are defined as arteries originating directly from the cerebral artery (*). Branches are defined as arteries originating from one common stem without any branches (▲). As shown in the figure, bilateral A1 has three trunks and five branches, while bilateral M1 has six trunks and 14 branches. In total, there are nine LSA trunks and 19 LSA branches. A1: The first segment of the anterior cerebral artery. M1: The first segment of the middle cerebral artery. LSAs: lenticulostriate arteries. ICA: internal carotid artery. ACA: anterior cerebral artery. MCA: middle cerebral artery.

Supplementary Table 1. The imaging parameters of 7.0-T MRI scanning

Sequence	TR (ms)	TE (ms)	FA (degree)	Resolution (mm^3)	FOV (mm^3)	TA†
MPRAGE	2200	3.29	8	0.70 × 0.70 × 0.70	224 × 203 × 180	5’06”
TOF-MRA	15	4.30	20	0.23 × 0.23 × 0.36	180 × 135 × 47	7’34”
FLAIR	14000	94	120*	0.43 × 0.57 × 3.00	220 × 199 × 117	7’58”
SWI	18	12	14	0.30 × 0.30 × 1.20	192 × 192 × 104	7’10”

* Refocusing flip angle. † With generalized autocalibrating partial parallel acquisition (GRAPPA), acceleration factor of two.

Abbreviation: TR: repetition time. TE: echo time. FA: flip angle. FOV: field of view. TA: time of acquisition. MPRAGE: magnetization-prepared rapid gradient-echo. TOF-MRA: time-of-flight magnetic resonance angiography. FLAIR: fluid-attenuated inversion recovery. SWI: susceptibility weighted imaging.

Supplementary Table 2. The ARWMC rating scale of basal ganglia for MRI

score	Basal ganglia lesions
0	No lesions
1	One focal lesion (≥ 5 mm)
2	> 1 focal lesion
3	Confluent lesions

Abbreviation: ARWMC: age-related white matter change. The following brain areas were used for rating: striatum, globus pallidus, thalamus, internal/external capsule, and insula.

Supplementary Table

Supplementary Table 3. Association between LSA measurements and MRI lesion load of the basal ganglia in CADASIL patients

Variables	Number of LIs		ARWMC scores		Number of CMBs	
	ρ (z)	P	ρ (z)	P	ρ (z)	P
Age [a]	0.259	0.082	0.314	0.033*	0.264	0.077
Sex [b]	-1.883	0.060	-0.778	0.437	-1.837	0.066
Disease duration [a]	0.044	0.792	0.107	0.521	0.101	0.547
History of smoking [b]	-1.646	0.100	-0.580	0.562	-0.221	0.825
History of alcohol consumption [b]	-2.424	0.015*	-0.387	0.699	-2.026	0.043*
Number of LSA branches [a]	-0.133	0.379	-0.128	0.398	-0.038	0.800
Proportion of discontinuous LSAs [a]	0.221	0.140	0.123	0.416	0.013	0.930
Length of LSAs [a]	-0.155	0.302	-0.180	0.231	-0.241	0.107

Abbreviation: LSA: lenticulostriate artery; MRI: magnetic resonance imaging; LIs: lacunar infarctions; ARWMC: age-related white matter change; CMBs: cerebral microbleeds. *Indicates a significant difference or a correlation between the two variables.

[a] Spearman rank correlation (ρ).

[b] Mann–Whitney U-tests (z).
